# Recent progress in targeting the sialylated glycan-SIGLEC axis in cancer immunotherapy

**DOI:** 10.20892/j.issn.2095-3941.2023.0046

**Published:** 2023-05-03

**Authors:** Yingyan Yu, Wenjie Peng

**Affiliations:** 1 Department of General Surgery of Ruijin Hospital, Shanghai Institute of Digestive Surgery, and Shanghai Key Laboratory for Gastric Neoplasms, Shanghai Jiao Tong University School of Medicine, Shanghai 200025, China; 2 Shanghai Center for Systems Biomedicine, Shanghai Jiao Tong University, Shanghai 200240, China

**Keywords:** SIGLEC, sialylated glycan, glyco-immune checkpoint, high affinity SIGLEC-ligands, anti-SIGLEC antibodies

## Abstract

Malignant tumors are complex structures composed of cancer cells and tumor microenvironmental cells. In this complex structure, cells cross-talk and interact, thus jointly promoting cancer development and metastasis. Recently, immunoregulatory molecule-based cancer immunotherapy has greatly improved treatment efficacy for solid cancers, thus enabling some patients to achieve persistent responses or cure. However, owing to the development of drug-resistance and the low response rate, immunotherapy against the available targets PD-1/PD-L1 or CTLA-4 has limited benefits. Although combination therapies have been proposed to enhance the response rate, severe adverse effects are observed. Thus, alternative immune checkpoints must be identified. The SIGLECs are a family of immunoregulatory receptors (known as glyco-immune checkpoints) discovered in recent years. This review systematically describes the molecular characteristics of the SIGLECs, and discusses recent progress in areas including synthetic ligands, monoclonal antibody inhibitors, and Chimeric antigen receptor T (CAR-T) cells, with a focus on available strategies for blocking the sialylated glycan-SIGLEC axis. Targeting glyco-immune checkpoints can expand the scope of immune checkpoints and provide multiple options for new drug development.

## Introduction

Malignant tumors are complex structures composed of cancer cells and various microenvironmental cells^1,2^, more than 50% of which are tumor-associated macrophages^3^. Cross-talk between cancer cells and microenvironmental cells facilitates cancer development and metastasis. Therefore, to conquer cancer, the biological behavior of cancer cells and the components of the tumor microenvironment (TME) cells, which greatly enhance treatment efficacy, must be considered.

In recent years, oncologists have recognized the biological importance of the TME in the progression of malignancies, particularly immune cells, and have attempted to ameliorate the immunosuppressive microenvironment of cancers caused by immune checkpoints^4,5^. Several monoclonal antibodies have been developed to block the PD-1/PD-L1 and CTLA-4 immune checkpoints. According to clinical treatment reports, use of an immunotherapeutic paradigm instead of traditional cytotoxic drugs can effectively reactivate immune cells. Thus, immune checkpoint inhibitors not only protect healthy cells against non-specific killing, but also enable durable response or even cure in patients^6,7^. Anti-cancer immunotherapies are a promising approach that has brought hope to patients. However, only limited patients show positive responses to PD-1/PD-L1 blockade therapy, owing to the variable expression of PD-1/PD-L1 among human populations and the development of drug-resistance after treatment. To date, the mechanism of primary or secondary resistance is not well understood^8,9^. Additional immunoregulatory pathways, such as T cell immune checkpoints, are likely to exist^10^. Consequently, combination strategies have been developed to target multiple immune checkpoints to enhance treatment efficacy^11^. Among them, sialic acid (Sia)-binding immunoglobulin-like lectins (SIGLECs) have attracted substantial attention as a potential alternative^12^. Here, we summarize recent progress in targeting the sialylated glycan-SIGLEC axis for cancer immunotherapy.

## SIGLEC classification and molecular characteristics

**Figure 1:**
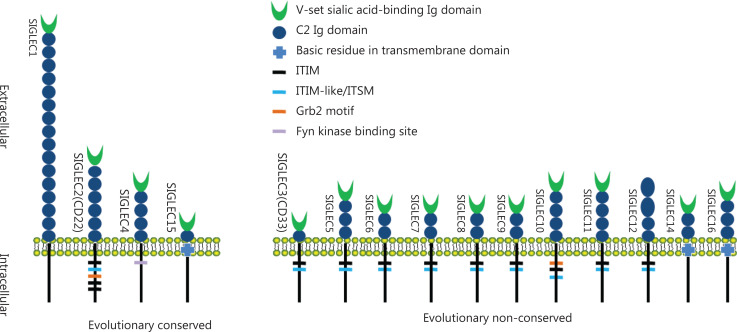
The 15 SIGLECs identified in humans. SIGLEC1, SIGLEC2, SIGLEC4, and SIGLEC15 are evolutionarily conserved, and the others are evolutionary non-conserved. SIGLEC1 is the longest SIGLEC without intracellular signaling motif, and human SIGLEC12 has lost the ability to bind Sias.

SIGLECs belong to the immunoglobulin superfamily, and are expressed on most immune cells. To date, 15 members of SIGLECs have been identified in humans. According to sequence similarity and evolutionary conservation, SIGLECs are classified into 2 categories. The first category is highly conserved among multiple vertebrate lineages and has low sequence similarity, and comprises SIGLEC1 (CD169, sialoadhesin), SIGLEC2 (CD22), SIGLEC4 (myelin associated glycoprotein, MAG), and SIGLEC15 (CD33L3). The second category lacks evolutionary conservation (i.e., has been identified in humans but not mice) and comprises the SIGLEC3 (CD33) related SIGLECs (CD33rSIGLECs), comprising SIGLEC3, SIGLEC5 (CD170), SIGLEC6 (CD327), SIGLEC7 (CD328), SIGLEC8, SIGLEC9 (CD329), SIGLEC10, SIGLEC11, SIGLEC12, SIGLEC14, and SIGLEC16^13–15^. The extracellular structure of SIGLECs consists of 1–16 Ig constant-2 set (C2) domains with an additional Ig variable set (V-set) domain at the N terminus, which is responsible for binding sialylated glycan (sialoside) ligands (**Figure 1**). In the cytoplasmic domain, most CD33rSIGLECs contain either an immunoreceptor tyrosine-based inhibitory motif (ITIM) or immunoreceptor tyrosine-based switch motif (ITSM). After binding sialoside ligands, the ITIM or ITSM recruits SRC homology region 2 domain-containing tyrosine phosphatase-1 and -2 (SHP-1 and SHP-2), and inhibits the activation of tyrosine kinase, thereby participating in immunosuppressive regulation. Several SIGLECs, such as SIGLECs 14, 15, and 16, have positively charged amino acid residues in their transmembrane domains, which interact with DAP12 (also known as transmembrane immune signaling adaptor TYROBP) on immune cells. The intracellular domain of DAP12 contains an immunoreceptor tyrosine-based activation motif (ITAM), which activates spleen tyrosine kinase (SYK) and further catalyzes a downstream immune cascade. Thus, DAP12-paired SIGLECs may participate in the activation of immune cells^16^.

SIGLECs are expressed on both innate and adaptive immune cells, such as monocytes, neutrophils, natural killer (NK) cells, and B cells. A recent article has indicated that adaptive immune cells such as T lymphocytes also express SIGLECs. Vuchkovska et al.^17^ have reported that SIGLEC5 is expressed on most activated T cells after antigen receptor stimulation, whereas SIGLEC5 overexpression abrogates the activation of NFAT and AP-1 induced by antigen receptor. The SIGLECs on human or murine leucocytes have diverse functions. Cells expressing SIGLECs are listed in **Table 1**.

**Table 1:** Expression spectrum of SIGLECs on human or murine cells

SIGLECs	Other names	Expressing cells	Refs
SIGLEC1	CD169	Macrophage, Dendritic cell	^14,18,19^
SIGLEC2	CD22	B cell, cDC*, Mast cell	^14,18^
SIGLEC3	CD33	Diverse myeloid-derived cells, NK cell, T cell	^14,18,20^
SIGLEC4	MAG	Oligodendrocyte, Schwann cell	^14,18^
SIGLEC5	CD170	Diverse myeloid-derived cells, T cell, B cell	^14,17,18,21,22^
SIGLEC6	CD327	Trophoblast, Mast cell, Basophil, B cell, Myeloid leukemia	^14,17,18,23^
SIGLEC7	CD328	Diverse myeloid-derived cells, NK cell, T cell	^14,18,24,25^
SIGLEC8	–	Eosinophil, Basophil, Mast cell	^14,18^
SIGLEC9	CD329	Diverse myeloid-derived cells, T cell, NK cell	^14,18,26^
SIGLEC10	–	Macrophage, NK cell, Eosinophil, B cell, T cell	^14,18,27^
SIGLEC11	–	Microglia, Macrophage, Ovarian stromal cell	^14,18,28^
SIGLEC12	Pseudogene	Macrophage, Unknown	^14,18,29^
SIGLEC14	–	Diverse myeloid-derived cells	^14,18,30^
SIGLEC15	CD33L3	Macrophage, Osteoclast	^14,18,31^
SIGLEC16	–	Macrophage, Microglia	^14,18,21^
mSiglec1**^#^**	mCD169	Macrophage, Dendritic cell	^14,18,19,32^
mSiglec2	mCD22	B cell, cDC*, Mast cell	^14,18,33^
mSiglec4	mMAG	Oligodendrocyte, Schwann cell	^14,18,34^
mSiglec15	mCD33L3	Macrophage, Osteoclast	^14,18,31^
mSiglec3	mCD33	Neutrophil, Macrophage, Microglia	^35,36^
mSiglecE	Homolog of SIGLEC9	Diverse myeloid-derived cells, NK cell, Dendritic cell	^37–42^
mSiglecF	Homolog of SIGLEC8	Immature cells of myeloid lineage, Eosinophil, Neutrophil	^35,43–49^
mSiglecG	Homolog of SIGLEC10	Eosinophil	^34,43,44^
mSiglecH	Possible human homolog of SIGLEC14 and SIGLEC16?	Plasmacytoid dendritic cell (pDC), Macrophage	^43,50–52^

^#^The “m” prefix indicates murine origin. *myeloid-derived DC.

## Natural ligands of SIGLECs

**Figure 2:**
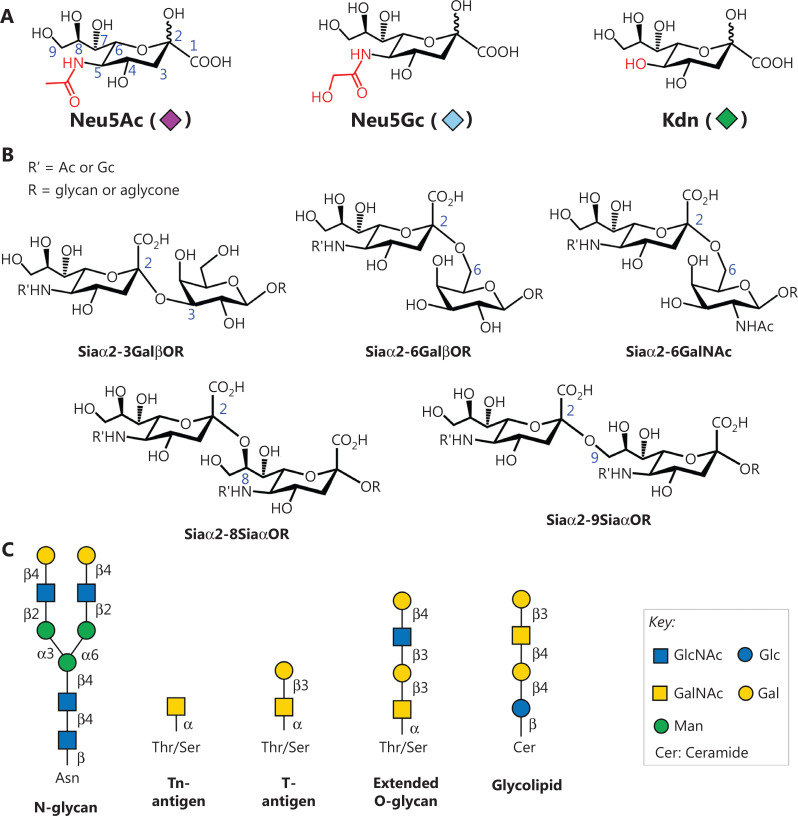
Diversity of sialoside structures. (A) Chemical structures of Neu5Ac, Neu5Gc, and Kdn. (B) Common linkage types of sialosides. (C) Underlying glycan backbones for sialylation, including glycoproteins (N-/O-glycan, Tn-, and T-antigen), as well as glycolipids. N-glycan is covalently attached to the amide side chain of the asparagine (Asn) residue, whereas O-glycan is attached to the hydroxyl groups of threonine/serine (Thr/Ser). Glycolipid is linked to the C-1 hydroxyl group of the ceramide. Structures are presented with SNFG symbol nomenclature (https://www.ncbi.nlm.nih.gov/glycans/snfg.html).

Sias are enriched on the surfaces of mammalian cells, bacteria and viruses, as well as on mucin proteins produced by cancer cells^53,54^. Sias are a family of sugar derivatives comprising a nine-carbon backbone with a carboxyl group at the C-1 position. The most common Sias in the mammalian glycome are N-acetylneuraminic acid (Neu5Ac), N-glycolylneuraminic acid (Neu5Gc), and the deaminated neuraminic acid 2-keto-3-deoxy-D-glycero-D-galacto-nononic acid (Kdn) (**Figure 2A**)^55^. Sias are frequently attached to the penultimate galactose (Gal) or N-acetyl galactosamine (GalNAc) residue through either an α2,3- or an α2,6-linkage. Sias can conjugate to the C-8 or C-9 positions, thus forming α2,8- or α2,9-linked sialosides (**Figure 2B**). Sialylation, an important glycosylation reaction, is accomplished by the transfer of Sias to the underlying glycan chain by a combination of cytidine monophosphate-Sia synthetases (CMP-Sia synthetases, CSSs) and sialyltransferases (STs). The linkage types are cell- and tissue-specific, and are dynamically regulated by the expression patterns of STs. Sialylated glycans are frequently attached to proteins (N-/O-linked glycoproteins) and lipids (glycolipids) involved in various biological processes, such as pathogen recognition, inflammation, immune responses, and cancer development (**Figure 2C**).

Owing to the attachment to the non-reducing end of glycan chains, Sias serve as ligands for certain cell membrane receptors, including SIGLECs^56^. However, SIGLECs have distinct binding specificity depending on the linkage type of Sias and the underlying sugar. A conserved arginine residue in the V-set domain is believed to ligate the carboxylate group of Sias *via* a salt bridge^57^. When essential arginine is mutated, Sia recognition ability is lost^58^. Further contacts have been observed between SIGLECs and the 4-OH, 5-NAc, and glycerol side chain of Sias. A variable C-C′ loop in the binding site is responsible for recognizing the underlying glycan^59^. Through interaction with the sialoside ligand, SIGLECs can distinguish “self” and “non-self” molecules, thus preventing unwanted inflammatory responses under homeostatic conditions.

The natural ligands of SIGLECs are sialylated glycans. However, recent studies have shown that lipophilic molecules and proteins mediate binding to SIGLEC receptors in a Sia-independent manner. Suematsu et al.^60^ have reported that fungal alkanes and triacylglycerols extracted from Trichophyton show ligand activity for SIGLEC5 and SIGLEC14. The lipophilic ligands suppress interleukin-8 (IL-8) production in SIGLEC5-expressing human monocytic cells, whereas the endogenous lipids induce IL-8 production in SIGLEC14-expressing human monocytic cells. These findings suggest that lipophilic ligands modulate innate immune responses, thus expanding understanding of the biological functions and importance of SIGLECs in innate immunity. In addition, Fong et al.^61^ have found that secreted heat-shock protein 70 (HSP70) acts as a ligand for SIGLEC5 and SIGLEC14, thus inducing either anti-inflammatory signal or pro-inflammatory signals, respectively. Moreover, Nizet and co-workers^62^ have demonstrated that human neonatal pathogen group B *streptococcus* engages SIGLEC5 and SIGLEC7^63^ *via* β protein, thus impairing human leukocytes, increasing bacterial resistance to neutrophil phagocytosis, and suppressing the pyroptosis activity of NK cells. A recent study has suggested that SIGLEC10 interacts with both amino acids and sialic acids of CD24, a protein overexpressed on tumor cells, thus inducing tumor immune escape^64^.

## Association of SIGLECs with cancers

Cancer development is regulated by the crosstalk between cancer cells and other components in the TME, such as cancer-associated fibroblasts, blood vessels, and immune cells. Although numerous immune cells are recruited to the local TME for targeting cancer cells, these abilities are inhibited by cancer-derived suppressive signals. Under suppressive conditions, immune effector cells, such as macrophages, dendritic cells, and T cells, do not have anti-cancer activity but instead facilitate cancer development.

The abnormal expression of some STs in cancer cells significantly affects Sia content and type. For example, a change in ST6GALNAC4 expression has been found to increase the content of disialyl-T antigen [Neu5Acα2,3Galβ1,3(Neu5Acα2,6)GalNAcα–]^65^. Moreover, hypoxia up-regulates the expression of both STs and the transporter SLC17A5, which transports external Sias into cells^66,67^. Thus, the cancer cell surface is covered by a dense layer of sialylated glycans, such as polysialic acid, sialylated Lewis antigens, and sialylated Tn/T antigens. Aberrant sialylation is associated with cancer progression and metastasis, and is a hallmark of several cancers including those of the lung, breast, pancreatic, and prostate^68^. These tumor-associated sialosides have been identified as biomarkers for certain cancers, and used for cancer diagnosis and monitoring. Among them, CA19-9 (also called carbohydrate antigen 19-9 or sialylated Lewis A antigen) is the most commonly used serum marker for pancreatic cancer diagnosis^69^.

Overexpressed sialosides on cancer cells interact with SIGLECs on immune cells providing an immunosuppressive TME just like the PD-1 does. Therefore, in recent years, SIGLECs have become a new target of anti-cancer immunity^70,71^. Stanczak et al.^12^ have reported the upregulation of SIGLECs including SIGLEC9 on tumor-infiltrating T cells from non-small cell lung cancer, colorectal cancer, and ovarian cancer. SIGLEC9-expressing T cells in patients with non-small cell lung cancer correlate with diminished survival, whereas SIGLEC9 polymorphisms are associated with the risk of developing lung and colorectal cancer. Targeting the sialoside-SIGLEC pathway increases anticancer immunity *in vitro* and *in vivo*. Moreover, Zhang et al.^72^ have reported that gastric cancer-specific exitrons significantly increase the expression of PD-1, SIGLEC1, SIGLEC2, SIGLEC3, and SIGLEC7 with high neoantigen load. The exitrons are clinically relevant to sex, age, Lauren classification, tumor stage, and prognosis. Wang et al.^73^ have constructed a comprehensive immune scoring system including 6 immunosuppressive genes (NECTIN2, CEACAM1, HMGB1, SIGLEC6, CD44, and CD155) to improve prognosis after adjuvant chemotherapy in gastric cancer by supplementing TNM staging. In addition, an interaction of SIGLEC7 and SIGLEC9 from myeloid cells with the elevated Sia in cancer cells has been found in a pancreatic cancer study^70^.

In our recent studies, we have analyzed the pangenomic characteristics of gastric cancer and identified a set of genes (GSTM1, ACOT1, SIGLEC14, and UGT2B17) with high-frequency absence variation at the whole genome level^74–76^. Through comparison with whole genome sequencing data for multiracial populations in public databases, we determined that the frequency of absence of the above 4 genes (41%–71%) in the gastric cancer population was much higher than that in European and American healthy populations (4.6%–46%). The absence of SIGLEC14 was first proposed in gastric cancer^76^. Because SIGLEC14 is an innate immune cell activation receptor, the integrity of the SIGLEC14 gene provides a molecular basis for ensuring the M1 polarization of macrophages or tumor-arresting polarization of neutrophils. Deletion of this gene in cancer is expected to worsen the tumor immunosuppressive microenvironment. A bioinformatic analysis of lung adenocarcinoma has indicated that the expression levels of SIGLEC3, SIGLEC5, SIGLEC7, SIGLEC9, SIGLEC11, and SIGLEC14 correlate with macrophage, neutrophil, and dendritic cell infiltration^77^.

## Strategies to block the sialoside-SIGLEC axis

The above studies have indicated that SIGLECs are involved in the immune evasion of cancers and are potential targets to alleviate the immunosuppressive TME in cancer immunotherapy. In the SIGLEC family, 8 members, SIGLEC3, SIGLEC5, SIGLEC6, SIGLEC7, SIGLEC8, SIGLEC9, SIGLEC10, and SIGLEC11, contain immunosuppressive functional domains in their intracellular domains, which are similar to PD-1^10,17^. Sequence alignment studies have demonstrated that PD-1 shares conserved amino acids in the ITIM and ITSM domains with SIGLEC5, SIGLEC7, and SIGLEC9. The interaction of SIGLECs with sialoside ligands results in inhibitory signaling as does the interaction between PD-1 and PD-L1^10^. Similarly to PD-1 based immunotherapies, blockade of the sialoside-SIGLEC axis provides benefits in cancer treatment.

### Multivalent presentation of natural ligands for targeting SIGLECs

Given that SIGLECs are glyco-immune checkpoints, the ligands or monoclonal antibodies targeting SIGLECs have therapeutic potential. Generally, natural sialosides on glycoproteins and glycolipids exhibit weak monovalent binding affinity toward SIGLECs (Kd = 0.1–3 mM), and this affinity can be increased by presentation of multiple copies to cluster of the SIGLECs^78^. To mimic the multivalent presentation of sialosides on the cell surface, researchers have prepared libraries of sialosides immobilized on glass slides (sialoside microarrays). Through high-throughput screening with the sialoside microarrays, natural ligands for SIGLECs have been identified (**Table 2**)^56^. As the sialylated glycans on traditional biochips cannot fully recapitulate their conformations on the cell surface, and the arrays are expensive, a mammalian living cell screening system has been developed^79^.

**Table 2:** Developed synthetic ligands for corresponding SIGLECs

SIGLECs	Natural ligands	High affinity synthetic ligands	Refs
SIGLEC1	Neu5Acα2,3LacNAc Modest	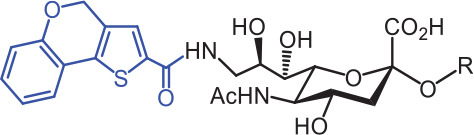 **^TCC^Neu5Ac (1)** (R = α2,3-LacNAc, IC_50_ = 0.38 μM)	^87^
hCD22*	Neu5Acα2,6LacNAc Strong	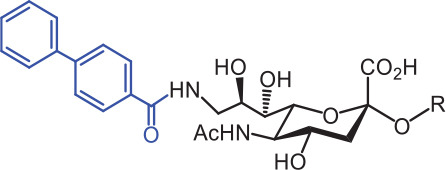 **^BPC^Neu5Ac (2)** (R = α2,6-LacNAc, IC_50_ = 0.20 μM)	^99^
		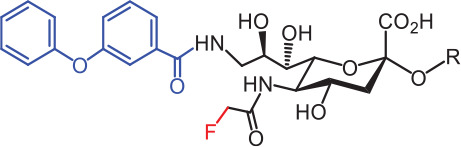 **^MPB^Neu5AcF (3)** (R = α2,6-Lac, IC_50_ = 0.20 μM)	^100^
mCD22**	Neu5Gcα2,6LacNAc Strong	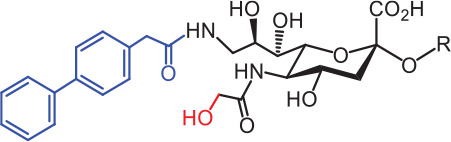 **^BPA^Neu5Gc (4)** (R = α2,6-LacNAc, IC_50_ = 0.80 μM)	^99^
hCD33***	Neu5Acα2,6LacNAc Weak	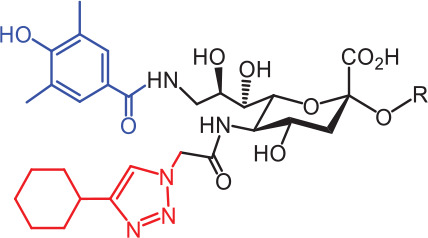 **(5)** (R = α2,6-Lac, IC_50_ = 11.00 μM)	^100^
SIGLEC7	Neu5Acα2,8Neu5Acα2,3LacNAc Strong	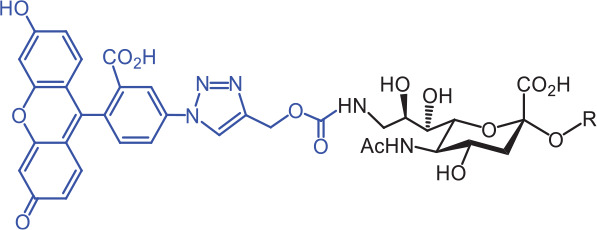 **^FTMC^Neu5Ac (6)** (R = α2,6-Lac, unknown affinity)	^89^
SIGLEC9	Neu5Acα2,3Galβ1,4(Fucα1,3)-(6-*O*-SO_3_)GlcNAc Strong [Ref. 101]	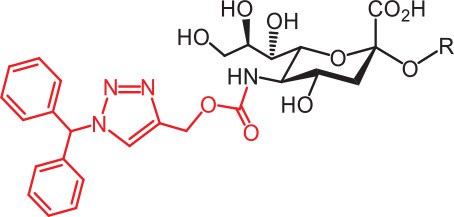 **(7)** (R = α2,6-Lac, unknown affinity)	^102^
SIGLEC15	Neu5Acα2,6GalNAcαThr/Ser (To be further evaluated) [Ref. 103]	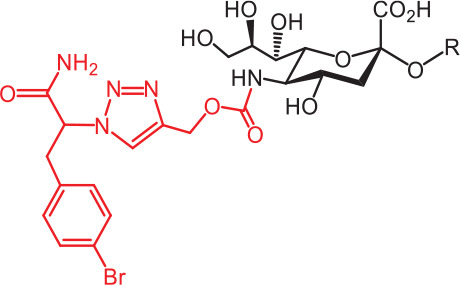 **(8)** (R = α2,6-LacNAc, unknown affinity)	^90^

LacNAc, Galβ1,4GlcNAc; Lac, Galβ1,4Glc; IC_50_, half maximal inhibitory concentration. *hCD22, human SIGLEC2/CD22. **mCD22, mouse SIGLEC2/CD22. ***hCD33, human SIGLEC3/CD33.

Physiologically, SIGLECs are masked by endogenous cis-ligands, thus aiding in maintenance of cell homeostasis; however, malignant cells show elevated interaction with inhibitory SIGLECs through hypersialylation, and dampened immune surveillance^80,81^. To block the sialoside-SIGLEC axis, natural sialosides have been incorporated into various polymeric scaffolds to mimic the multivalent presentation of sialosides on glycoproteins and glycolipids^82–85^. Glycopolymers with a high density of Sia moieties can outcompete the natural sialosides in cancer cells for SIGLEC binding. Thus, sialoside glycopolymers can be used as inhibitors to perturb SIGLECs. To validate early models of hypersialylation-mediated immunoevasion, Bertozzi and coworkers^82^ have incorporated sialoside-functionalized glycopolymers onto cancer cell surfaces. The results suggest that hypersialylation of cancer cells elicits NK inhibition, and SIGLEC7 can tune the cytotoxicity activation of NK cells according to cancer cell sialylation status^82^. These results indicate that SIGLEC7 may be a potential therapeutic target for cancer therapy.

Because SIGLECs bind natural ligands with overlapping specificity and lower affinity than synthetic ligands, their regulatory mechanisms may be misinterpreted. Therefore, high affinity synthetic ligands with better specificity for SIGLECs must be developed.

### Development of synthetic ligands for SIGLECs

In the past 20 years, various strategies have been used to introduce novel substituents to Sias as synthetic ligands, thus increasing binding affinity to SIGLECs in the sub-micromolar range (**Table 2**)^86^.

Because of the lack of an intracellular signaling motif, SIGLEC1 (sialoadhesin, Sn) is an ideal receptor for targeted delivery of antigens to macrophages, thereby eliciting a robust humoral response. The crystal structures of murine Sn have been determined, thus providing structural insights into the key features of Sia recognition. A high affinity and specificity ligand ^TCC^Neu5Ac sialoside (**1**), with sub-micromolar binding affinity (IC_50_ = 0.38 μM), has been developed^87^. Through screening with a sialoside analog microarray, several high affinity ligands for SIGLEC2/CD22 have been identified, such as the ^BPC^Neu5Ac (**2**) and ^MPB^Neu5AcF (**3**) sialosides for human CD22, and ^BPA^Neu5Gc (**4**) sialoside for murine CD22^88^. Through the same strategy, ^FTMC^Neu5Ac (**6**) has been discovered as a high affinity ligand for SIGLEC7, an inhibitory receptor on NK cells^89^. Moreover, cell-based glycan arrays have been developed to directly probe interactions of glycans with glycan-binding protein on the Chinese hamster ovary cell surface. With this platform, high-affinity glycan ligand **8** was discovered for SIGLEC15^90,91^. A panel of synthetic ligands has been developed. Examples are listed in **Table 2**, including SIGLEC1, SIGLEC2, SIGLEC3, SIGLEC7, SIGLEC9, and SIGLEC15.

However, these synthetic ligands alone remain insufficient to unmask the binding sites of endogenous target cell cis-ligands on SIGLECs. Targeting specific SIGLEC on cells requires multivalent presentation of high affinity ligands on various scaffolds, including nanoparticles and polymers^92–94^. For example, liposomal nanoparticles coated with the high affinity CD22-ligand BPC-Neu5Ac sialoside have been generated to target human malignant B cells^92^. After binding and endocytosis into acidic endosomes, liposomes are broken, and the encapsulated toxins are released, thus achieving CD22-dependent cytotoxicity in *in vitro* and *in vivo* studies. In addition, through metabolic engineering or a chemoenzymatic approach, the high affinity CD22-ligand MPB-Neu5Ac has been incorporated on NK-92 cells and found to enhance anti-tumor activity^95,96^. Glyco-engineered NK-92 cells exhibit CD22-dependent cytotoxicity to lymphoma cell lines and primary lymphoma cells from human patients. In recent studies, Bertozzi and coworkers^97^ have incorporated the SIGLEC9 high affinity ligand into a synthetic polypeptide. The artificial glycopeptide serves as a membrane-tethered cis-binding agonist that inhibits macrophage phagocytosis and induces neutrophil apoptosis^98^.

The above studies have highlighted the potential applications of synthetic SIGLEC ligands as immune modulators with great medicinal value in cancer treatment.

### Progress in monoclonal antibodies for SIGLECs

Because cancer cells inhibit immune cell activity and evade immunosurveillance *via* the sialoside-SIGLEC axis, scientists have developed monoclonal antibodies targeting these inhibitory SIGLECs. By immunizing mice with SIGLEC9-encoding DNA and SIGLEC9 protein, Choi et al.^42^ have developed the high specificity and functionality monoclonal antibody (8A1E9) against SIGLEC9. The humanized antibody shows anti-tumor immune activity toward ovarian cancer *in vitro* and *in vivo*. Similarly, Cyr et al.^104^ have developed an anti-SIGLEC6 monoclonal antibody achieving highly potent and specific elimination of SIGLEC6 positive leukemic and healthy B cells, thus indicating the potential for cancer immunotherapy.

SIGLEC15 has recently been identified as a critical immune suppressor. Chen and coworkers^31^ have identified the SIGLEC15 immune suppressor through a genome-scale T-cell activity array. They have found that SIGLEC15 is broadly upregulated on human cancer cells and tumor-infiltrating myeloid cells. Importantly, the expression of SIGLEC15 is mutually exclusive to PD-L1. By binding unknown ligands, SIGLEC15 suppresses antigen-specific T-cell responses *in vitro* and *in vivo*. Genetic ablation or antibody (clone m03) blockade of SIGLEC15 amplifies anti-tumor immunity in the TME and inhibits tumor growth in some mouse models^31^. Xiao et al.^105^ have reported a monoclonal antibody against SIGLEC15 (S15-4E6A), and evaluated its antitumor effectiveness and modulatory role in macrophages *in vitro* and *in vivo*. They have found that S15-4E6A promotes macrophage M1 polarization while inhibiting M2 polarization both *in vitro* and *in vivo*, and exerts an efficacious tumor-inhibitory effect on lung adenocarcinoma cells and xenografts. He et al.^106^ have developed a monoclonal antibody against SIGLEC15 (3D6), which blocks SIGLEC15-mediated suppression of T cell and moderately prevents tumor growth. Wu et al.^107^ have conducted monoclonal antibody screening on SIGLEC15 and have found that the 3F1 clone antibody has high receptor blocking activity and significantly reverses the inhibitory effect of SIGLEC15 on lymphocyte proliferation. In mouse experiments, the 3F1 monoclonal antibody has shown significant antitumor efficacy when applied alone or in combination with the Erbitux drug. These results have demonstrated that SIGLEC15 is a potential target for normalizing tumor immunity as an alternative to anti-PD-1 therapy.

AMG 330 is a dual specific antibody for CD3 and SIGLEC3/CD33. CD33 is frequently expressed on the surfaces of blasts and leukemic stem cells in acute myelogenous leukemia. AMG 330 binds with low nanomolar affinity to CD33 and CD3ε of both human and cynomolgus monkey origin. In an *ex vivo* experiment, AMG 330 has been found to mediate autologous depletion of CD33-positive cells from cynomolgus monkey bone marrow aspirates. Thus, AMG 330 is a potential anti-tumor reagent for acute myelogenous leukemia^108^.

The above studies have indicated that antibodies against SIGLEC checkpoints provide an alternative treatment for patients with cancer refractory to the well-known PD-L1/PD-1-targeting therapies.

Because of the selective expression and endocytosis properties, SIGLECs can be directly targeted to deliver toxic cargo into hematopoietic cancer cells. In 2000, Mylotarg (Gemtuzumab ozogamicin from Pfizer), an anti-CD33 antibody-calicheamicin conjugate, was the first antibody-drug conjugate approved by the U.S. Food and Drug Administration (U.S. FDA). Mylotarg was developed for the treatment of acute myeloid leukemia but was withdrawn because of its high toxicity and low efficacy in 2010. However, with altered dosing, Mylotarg regained approval for treatment of acute myeloid leukemia in 2017^109–111^. Similarly, in 2017, another antibody-drug conjugate drug, Besponsa (Inotuzumab ozogamicin, Pfizer, NCT01564784), was approved by the U.S. FDA to treat CD22-positive B-cell precursor acute lymphoblastic leukemia^112^.

### Progress in dual functional drugs for desialylation-targeted therapy

During cancer development, tumor cells acquire the ability to evade immunosurveillance; the sialoside-SIGLEC axis between cancer cells and immune cells in the TME plays an important role in this evasion. However, the binding of SIGLECs to Sias is dynamic and reversible. Sialidases (called neuraminidases, NEUs) are enzymes that cleave the terminal Sia resides from glycolipids and glycoproteins, and are involved in several human pathologies such as neurodegenerative disorders, cancers, and infectious and cardiovascular diseases^113^. The four types of mammalian sialidases, encoded by different genes, are NEU-1, NEU-2, NEU-3, and NEU-4. Mucins (MUCs) are the major substrates of sialidases^114^. Therefore, sialidase dissociates SIGLECs bound to their ligands. By chemically coupling recombinant sialidases to trastuzumab, human epidermal growth factor receptor 2 (HER2)-specific antibody-sialidase conjugates have been constructed to desialylate tumor cells in a HER2-dependent manner, thus disrupting the sialoside-SIGLEC axis and enhancing antibody-dependent cell-mediated cytotoxicity^10,115^. Single-cell RNA sequencing has revealed that desialylation repolarizes tumor-associated macrophages and enhances the efficacy of immune checkpoint blockade^116^. Antibody-sialidase conjugates are thus a promising modality for glyco-immune checkpoint therapy.

Macrophages are important innate immune cells that provide the first line of defense against the invasion of harmful foreign molecules (immune defense) and autologous damaged or dead cells (immune surveillance). Unlike T and B lymphocytes, macrophages can kill foreign microorganisms and tumor cells non-specifically. The polarization status and regulatory mechanisms of macrophages have become major research fields. Macrophages in the TME can polarize in 2 directions depending on external stimuli: M1-type polarization (classical activation of macrophages) and M2-type polarization (alternative activation of macrophages), similarly to Th1 and Th2 activation of T lymphocytes. M1-polarized macrophages are pro-inflammatory cells, which secrete inflammatory factors such as TNF-α and IL-1β, and extend pseudopodia for active phagocytosis. M2-polarized macrophages secrete cytokines such as IL-10 and TGF-β, which induce the production of Treg cells in the TME and promote tumor growth^117–119^.

Similarly, neutrophils can have either tumor-arresting or tumor-promoting functions^40^. Recently, new knowledge has been highlighted regarding tumor-infiltrating neutrophils. Xue et al.^120^ have found that CCL4 and PD-L1 positive tumor-associated neutrophils have a tumor-promoting function in liver cancer. Moreover, the cytokines and chemokines secreted by neutrophils influence innate and adaptive immunity. IL-12, TNF-α, GM-CSF, CXCL10, CCL7, CCL2, and CCL3 are proinflammatory cytokines that serve as T cell and macrophage chemo-attractants. However, CCL17 and CXCL14 are pro-tumor cytokines^121^. Ligands on pathogens or tumor cells bind SIGLEC9 on neutrophils and limit neutrophil activation^122^. Thus, CD33rSIGLECs have been recognized as negative regulators of neutrophils. In addition, aberrant sialoglycans on the surfaces of tumor cells can shield potential tumor antigen epitopes and escape recognition, thereby suppressing immunocyte activation. Desialylation on tumor cells can present tumor antigens with Gal/GalNAc residues and thus overcome glyco-immune checkpoints. Huang and colleagues^123^ have explored whether vaccination with desialylated whole-cell tumor vaccines (ID8 vaccine) might trigger anti-tumor immunity in ovarian cancer. A desialylated tumor cell vaccine has been found to promote anti-tumor immunity and provide a strategy for ovarian cancer immunotherapy in a clinical setting^123^.

### Chimeric antigen receptor T cell (CAR-T) and other approaches

CAR-T approach uses a genetically modified T cell receptor with improved recognition of specific cancer cell antigens and tumor cell killing. CD19 is by far the most targeted biomarker in cancer immunotherapy^124^. CD19 CAR-T has been used for B-cell acute lymphoblastic leukemia or lymphoma therapy. However, relapse occurs in some cases. Thus, CD22/SIGLEC2 CAR-T and CD33/SIGLEC3 CAR-T were developed for the treatment of refractory leukemia or lymphoma^125^. A clinical trial has investigated patients with relapsed/refractory large B-cell lymphoma after CD19/22 dual-targeting CAR-T (AUTO3) plus pembrolizumab for relapsed/refractory large B-cell lymphoma (NCT03289455) and observed an overall response rate of 66% (48.9%, CR; 17%, PR)^126^. Because CD22 is restricted to surfaces of B cells and B lymphoma cells, it is a commonly used target for the treatment of autoimmune diseases and B-cell malignancy. Currently, immunotherapy drugs targeting CD22 include monoclonal antibody drugs, antibody-drug conjugates, and CAR-T therapies. In addition, SIGLEC6 has been reported as a novel target for CAR T-cell therapy in acute myeloid leukemia^23^.

Given that hypersialylation of cancer cells together with significant upregulation of ST contributes to cancer progression and drug resistance^127,128^, scientists have designed and constructed long-circulating, self-assembled core-shell nanoparticles carrying a transition state-based ST inhibitor, which inhibits sialoglycans in various cancer cells^129^. Recently, the Bertozzi group^41^ has found that the MYC oncogene controls expression of the sialyltransferase ST6GALNAC4 and induces sialosides, which function as a “do not eat me” signal by engaging SIGLEC7 of macrophages, thus hindering cancer cell clearance. Therefore, ST6GALNAC4 is a potential enzyme target for small molecule-mediated immune therapy^41^. Recently, Wang and coworkers^80^ have found that classical conventional DCs from cancer patient samples have high expression of several inhibitory SIGLECs including SIGLEC7, SIGLEC9, and SIGLEC10. In subcutaneous murine tumor models, downregulation of the inhibitory mSiglecE receptor on cancer-associated DCs has been found to enhance priming of antigen-specific T cells and induce proliferation. The above studies reveal a potential new target to improve cancer immunotherapy^80^.

In addition, soluble SIGLECs can function as immunomodulatory molecules, because binding to sialoside ligands interferes with the interaction between membrane SIGLECs and ligands. For example, Tomioka et al.^130^ have found that transgenic mice expressing the soluble form of mSiglecE show significant suppression of MUC1-expressing tumor proliferation. Related therapeutic interventions might potentially alter the outcomes of certain diseases. Tumor-associated MUC1 binds SIGLEC9, thus mediating tumor cell growth and inducing negative immunomodulation. Ono et al.^131^ have proposed that soluble SIGLEC9 (sSIGLEC9) competitively inhibits the binding of MUC1 to the receptor SIGLEC9, thus conferring an antitumor benefit against MUC1-expressing tumors. Moreover, soluble SIGLEC14 in the blood has been found to dose-dependently suppress the pro-inflammatory responses of myeloid cells expressing membrane-bound SIGLEC14^132^.

## Conclusions

**Figure 3:**
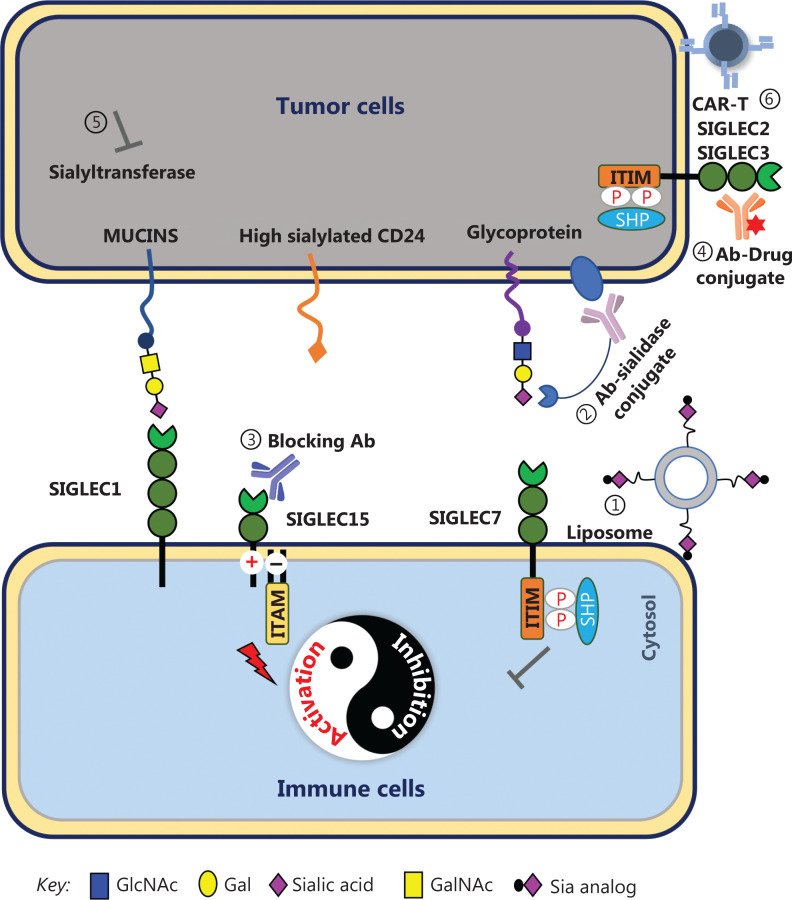
Strategies for targeting the sialylated glycan-SIGLEC axis. (1) Liposomal nanoparticles coated with high affinity ligands deliver anti-tumor drugs to lymphoma cells, which express SIGLEC2 or SIGLEC3. (2) Antibody-sialidase conjugates destroy the sialic acids on tumor cells and release the SIGLEC receptors. (3) Anti-SIGLEC antibodies block the specific sialoside-SIGLEC axis. (4) Antibody-drug conjugates target and are endocytosed into tumor cells by SIGLECs. (5) Sialyltransferase inhibitors decrease sialyltransferase expression. (6) SIGLEC-specific CAR-T increases the cytotoxicity of immune cells.

In summary, to overcome the immunosuppressive state of malignancies, scientists have developed various strategies to target the sialylated glycan-SIGLEC axis (**Figure 3**), although some strategies remain in the conceptual stage or pre-clinical research. As an alternative therapy or combination strategy with immune checkpoint inhibitors, targeting of the sialylated glycan-SIGLEC axis is expected to have a major role in cancer immunotherapy. At present, development of anti-SIGLEC drugs is rapidly progressing, including high affinity ligands, monoclonal antibodies, dual functional reagents of desialylation molecular targeted drugs, and CAR-T cells. The physiological roles of SIGLECs, a new generation of immune checkpoint, continue to expand and are expected to attract greater attention in cancer immunotherapy.
